# Intravenous Injection of Ammonia

**Published:** 1879-10

**Authors:** 


### INTRAVENOUS INJECTION OF AMMONIA.

Dr. Griswold, in the *Medical Record*, has an interesting article on this subject. He first made several experiments on dogs, using the ordinary aqua ammonia (containing ten per cent. of ammonia gas), diluting it with an equal bulk of water. He selected for experiment dogs in which the viscera had been exposed for purposes of vivisection, and had become exhausted from loss of blood and the depression attending the entrance of cold air into their thoracic and abdominal cavities. He waited in such a case until the heart had almost ceased to beat, its rhythm being disturbed, and its inefficient contractions no longer deserving to be called pulsations. He then injected into a convenient vein half a drachm of ammonia solution. After a period varying with the distance of the vessel selected from the heart, and with the rapidity of the circulation in the particular case, a marked change was observed. The heart had a moment before been dark and congested, its right cavities engorged, and the contraction of its fibres weak and uncertain. Suddenly the systole acquired new energy, which emptied the distended right ventricle into the lungs and filled the aorta with fresh oxygenated blood; the heart itself became right again as the new supply flowed in through the coronary arteries. The circulation was almost immediately re-established and the animal, if anæsthesia were not too complete, moved and showed signs of life. In the course of fifteen or twenty experiments he never failed to obtain the result described above. He has several times in Bellevue Hospital injected one drachm of ammonia solution into the veins of patients apparently moribund, and always succeeded in stimulating them much more powerfully than by other methods. On one occasion, a man came in a great hurry, having been notified that his brother was dying of phthisis. Notwithstanding his haste the sick man was already moribund and unconscious when he arrived. Pitying his disappointment of being too late for a few last words, the doctor injected a drachm of ammonia solution in the cephalic vein of the patient. In five minutes the man, who had appeared almost dead, was sufficiently restored to speak, and half an hour elapsed before he again became unconscious.

The doc'or mentions several cases in which the result was apparent, but one case especially is so remarkable that we will reproduce it in full.

Hester Mahar, aged forty-seven, Irish, single. Admitted to Bellevue Hospital April 29. On admission there was ascites, which had commenced a month before and was probably due to cirrhosis of the liver. Right pleural cavity nearly full of fluid, heart displaced to the left. No evidence of cardiac or renal disease. Patient very weak and compelled, from dyspnœa, to maintain a sitting posture. Abdomen tapped ; seven quarts and eight ounces of clear serum withdrawn. Patient much relieved. Stimulants and nutritous diet ordered.

May 1. Patient very weak. Does not seem to suffer very much from dyspnœa, though the right side is nearly full. Considered advisable to postpone thoracentesis until the patient is stronger.

May 3. Patient still very weak; dyspnœa not marked.

May 4. Called by nurse to see patient. Found her breathing very little; weakness seeming to obscure the expression of dyspnœa. Almost unconscious. Cannot be made to notice anything or swallow what is put on her lips. Fluids poured into her mouth run out again. Eyes vacant, pupils dilated, jaw fallen, tongue dry and brown. Thoracentesis performed with the assistance of three members of the house-staff. Ninety ounces of clear serum drawn off. During the operation, which lasted about twenty minutes, fifteen or twenty half-drachm doses of whisky were administered hypodermically. In spite of these efforts at stimulation, the pulse, which had before been weak, now disappeared entirely at the wrist. The impulse of the heart could scarcely be felt over the præcordia, and the respirations were shallow and ineffectual, not seeming adequate to the inflation of the lung just relieved from the pressure of the fluid. The condition of the patient was so unpromising that my colleagues of the house-staff, who had been assisting me, were of opinion that she was dying, and that further treatment was useless and even absurd. Expressing themselves to this effect, they left me, giving up the case in their own minds, and taking no further interest in the matter. While I was obliged to admit that the case was hopeless, judged by ordinary standards, and beyond the reach of ordinary stimulants, I could not help feeling that heroic measures were specially indicated. The source of trouble—fluid compressing a lung and displacing the heart—had been removed; if the patient could be stimulated to breathe deeply and profit by its disappearance, there seemed to be good reason to hope for her recovery.

Selecting a prominent superficial vein in the radial region, I exposed it by an incision through the skin. I then injected slowly into it a drachm of ammonia solution, taking care that the point of the hypodermic needle was free in the lumen of the vessel. This done, I placed my hand over the patient's heart and waited. In fifteen seconds, I felt a marked increase in the force of pulsation. In about two minutes there was a strong pulse of a hundred, which was plainly perceptible at the wrist. A minute later the patient sighed deeply; the color came back to her lips, her eyes moved and began to show signs of returning intelligence. On being urged, she swallowed without difficulty two ounces of strong egg-nog. After a few deep inspirations, she breated more regularly and easily; her pulse was strong and tense, ranging between 100 and 110. Half an hour afterward she was perfectly conscious, and reported herself comfortable, though weak. Pulse 90, regular strong. Respirations 26, easy and natural. Swallowed easily and willingly small quantilies of egg-nog. During the afternoon and evening, patient continued to improve. Pulse 80–90 and strong, respirations 20–30 and easy. Patient passed a good night, sleeping most of the time. Was bright and refreshed in the morning.

May 7. Steady improvement since last note. Sat up for two hours to-day, and ate a lamb chop with relish.

May 17. Patient sits up nearly every day, and is gaining strength.

N. B.—Improvement has been uninterrupted since the injection of ammonia. No depression has been observed following the stimulant action of that remedy, nor has there occurred an unpleasant symptom which could be attributed to it.

The case described seems to satisfactorily establish :

1. That the intravenous injection of ammonia is a prompt and powerful means of stimulation, acting efficiently in cases where other measures are of no avail.

2. That no bad effects follow its employment.

While the importance of the above deductions are obvious as a matter of general therapeutic interest, they seem to have a special significance in connection with those operations whose object is the removal of mechanical obstructions to respiration—I mean thoracentesis, and more particularly laryngotomy and tracheotomy. Thoracentesis is not, perhaps, very often an emergency; but laryngotomy and tracheotomy, done in cases of croup, oedema glottidis, generally fail to save life, because performed too late—the patient being too much exhausted to breathe in the air for which a new entrance has been made. Artificial respiration, hypodermics of whisky and ether, cold effusion, etc., are resorted to in vain in many instances—the machinery of life cannot be set in motion again, and the cases die for want of efficient stimulation. Now, would not the intravenous injection of ammonia, in connection with artificial respiration, save many of these patients? It being proved that the treatment is without danger and followed by no bad effect, this question should not long remain unanswered.

In conclusion, I would call attention to the fact that it is not easy to perform intravenous injection through the skin. The vein collapses under the necessary pressure, and the needle is apt either to stop short and not enter the vessel at all, or to transfix it and direct the injection into the cellular tissue beyond. The only safe method to pursue is to dissect down upon the vein and expose it; the needle may then be carefully introduced until the point is left free in the interior of the vessel.—*Buffalo Medical and Surgical Journal.*

